# Well-being profiles in adolescence: psychometric properties and latent profile analysis of the mental health continuum model – a methodological study

**DOI:** 10.1186/s12955-020-01332-0

**Published:** 2020-04-06

**Authors:** Melinda Reinhardt, Zsolt Horváth, Antony Morgan, Gyöngyi Kökönyei

**Affiliations:** 1 Institute of Psychology, ELTE Eötvös Loránd University, Budapest, Hungary; 2 Doctoral School of Psychology, ELTE Eötvös Loránd University, Budapest, Hungary; 3 Glasgow Caledonian University, London, UK; 4 SE-NAP2 Genetic Brain Imaging Migraine Research Group, Hungarian Academy of Sciences, Semmelweis University, Budapest, Hungary; 5 Department of Pharmacodynamics, Faculty of Pharmacy, Semmelweis University, Budapest, Hungary

**Keywords:** Well-being, Positive mental health, Mental health continuum model, Adolescents, Confirmatory factor Analysis, Exploratory structural equation modeling, Gender invariance, Latent profile analyses

## Abstract

**Background:**

The Adolescent Mental Health Continuum Short Form (MHC-SF) is a psychometrically valid tool to evaluate the domains of subjective well-being, but there is a lack of investigations which could distinguish subgroups with distinct subjective well-being profiles based on this measurement. Therefore, after testing the competing measurement models of the MHC-SF, our main aim was to identify subjective well-being profiles in a large adolescent sample.

**Methods:**

On a representative Hungarian adolescent sample (*N* = 1572; 51% girl; mean age was 15.39, SD = 2.26) confirmatory factor analyses (CFA) and exploratory structural equation modeling (ESEM) were used to test the factor stucture of the Adolescent MHC-SF. In addition, gender invariance of the best fitting model was also tested. Latent Profile Analyses (LPA) were conducted to reveal distinct subgroups and these profiles were then compared.

**Results:**

Results support the bifactor model of MHC-SF: the general and specific well-being factors which were invariant across gender. LPA yielded four subgroups, three of them have been theoretically hypothesized in previous works (i.e. flourishing, moderate mental health, languishing), but an emotionally vulnerable subgroup also emerged. Compared to the languishing group, this new subgroup demonstrated higher scores on prosocial behaviour, but had comparable level of loneliness and internalizing symptoms.

**Conclusions:**

Our results suggest that the MHC-SF is a reliable and valid instrument for assessing overall well-being and its components. In addition, the identification of young people to be at risk for low mental health may help us to tailor mental health promotion programs to their special needs.

## Background

The Mental Health Continuum Model (MHCM [1] is a theoretically well-grounded, complex mental health concept, derived from positive psychology. Keyes [2], who developed the MHCM framework, highlights that not everyone with low subjective well-being experiences psychopathology, accordingly positive mental health is related to, but different from mental illness. In this dual continua model three levels of mental health (1–3) and three states of mental illness (4–6) emerge: (1) flourishing (high mental health with low mental illness); (2) pure languishing (low mental health and low mental illness); (3) moderate mental health (average mental health with low mental illness); (4) flourishing and mental illness; (5) moderate mental health and mental illness, and (6) languishing together mental illness. According to the model, all three mental health states can exist in itself (1–3), but can also occur together with mental illness symptoms (4–6). In rare circumstances it is also possible that individuals with higher levels of subjective well-being (that is flourishing persons) simultaneously have mental illness (e.g., anxiety or stress) symptoms (4) [3].

Keyes [1] identified the components of mental health. Based on integration of earlier theories and research this multidimensional subjective well-being model rests on three foundations: emotional together with psychological and social well-being [4].

With this theoretical background Keyes [1] developed a questionnaire, the Mental Health Continuum Short Form (MHC-SF) to assess the three facets of well-being. The MHC-SF has adult [1, 5] and adolescent versions [6], has been translated into several languages and verified in numerous populations: among adolescents [6], adults [7], elderly adults [8]) or psychiatric patients [9] and in different cultures [10]. A number of studies have testified the psychometric properties of the questionnaire [5, 11–14].

Over the last decade or so, several studies have examined the factor structure of the MHC-SF. Originally these research used confirmatory factor analysis (CFA) to test competitive models (one-factor, two-factor, three-factor, bifactor solutions). However more recently, researchers have also applied exploratory structural equation modeling (ESEM), because it can provide more accurate factor intercorrelations. Outcomes of these analyses have been discordant. Some support the three-factor model (emotional, psychological, social well-being) both in adolescent [11, 12, 15–17] and in adult samples [5, 18]. Others studies, mainly those which used ESEM, alluded to a bifactor structure among adolescents [19, 20] and adults [21, 22] and other detected good fit for both three-factor and bifactor models on their adult data [13, 23]. In general, the one (unidimensional general well-being) and two factor (emotional well-being and psychological well-being with social well-being) solutions were not supported (except Machado and his colleagues in 2015 [24]). Results on the bifactor model are important, because they highlight that both individual well-being factors and when combined have a legitimate use and interpretation. Almost without exception full measurement invariance across gender [16, 25] and total [16] or partial invariance across age [20, 26] and cultural groups [27] were detected either in youth or adult samples.

Up until recently, there has been a few research that has studied adolescent samples in the context of the MHC-model [6, 11, 12, 15–17, 19, 20]. Given what is known about the relevance of the adolescence as a key developmental stage for growth and health in adulthood, these more recent studies are important [28]. Keyes [6] tested the MHC-SF including the three factor well-being model among 12–18 years old American adolescents in a national survey. The results showed, 56% of the adolescents were moderately mentally healthy, 38% of them were identified as flourishing, and a small proportion (6%) of them languishing. Flourishing adolescents showed the best psychosocial functions, while languishing ones recorded the most depressive symptoms and behavioural problems. Keyes [6] defined the three mental health groups using pre-defined (arbitrary) cut-off scores of the three subscales, but neither he, nor other scientists have tested how these groups fit to observed data.

The recently published studies focusing on the measurement of adolescent mental health using MHC-SF provide diverse samples across age range, sample size and study design [6, 11, 12, 15–17, 19, 20]. Moreover, apart from two studies [16, 19] all of the surveys used to capture Adolescent MHC-SF included Non-European samples [6, 11, 12, 15, 17, 20].

Based on our review of previous research on the MHCM, the first aim of this study was to verify the MHC model in a Central European representative adolescent sample covered the whole adolescence at all age levels.

On the other hand, we aimed to analyze the factor structure, as well as measurement invariance across gender of the Hungarian version of the MHC-SF. In this context our purpose was to compare the Hungarian results with previous studies, accordingly to take a stand on the discussion about the dimensionality of the MHC-SF.

A major aim of us was to investigate latent profiles of subjective well-being in a representative adolescent sample. Latent profile analysis is a technique that helps to identify homogeneous subgroups of participants. In relation to this our further goal was to feature the psychosocial characteristics of the different mental health profiles. To the best of our knowledge, no study has thus far targeted latent profile analysis (LPA) on the MHCM. Although Joshanloo in 2018 [29] identified underlying dimensions, three non-overlapping clusters with multidimensional scaling (MDS) on MHC-SF in a huge American college student sample, but his approach was item-oriented. Our analysis is a person-oriented solution, which allows us to focus on the profiles, the special characteristics of participants instead of testing a theoretical model [30]. Using LPA we can separate a large adolescent sample into classes based on their self-evaluation on items which refer to subjective well-being.

## Methods

### Participants and procedures

Students above the age of 11 from every primary and secondary school in the 21th District of Budapest were asked to participate in the research. Participants were invited to complete paper-based questionnaires in their classrooms with the supervision of trained principal investigators. No teaching staff were present. Participation in the study was voluntary and anonymous. Written informed consent was sought from all of respondents and one of their parents. One hundred fourteen parents refused the permission that their child participate in the study and 178 students were absent during data collection. One thousand six hundred twenty-five students completed the questionnaire. Following a review of missing data 53 people were excluded from the dataset. The final sample contained 1572 adolescents. Forty-nine percent of the sample were male (*N* = 770), 51% were female (*N* = 802). The mean age of the adolescents was 15.39 years (SD = 2.26), with an age range of between 11 and 20. The study was ethically approved by the Institution of Review Board of Eötvös Loránd University and the work was conducted in accordance with the Declaration of Helsinki.

### Measures

#### Demographics

Participants provided data on age, gender, school performance, loneliness, and perceived financial circumstances of their family.

#### Positive mental health

The 14 item-long Adolescent MHC–SF [6] covers three basic subjective well-being domains: 3 items refer to emotional, 6 items to psychological and 5 items to social aspects of well-being. Respondents rated the frequency of each feeling in the past month on a Likert-type scale from never (0) to every day (5). The Hungarian version of the MHC-SF was developed with agreement of the original author, Corey L. M. Keyes.

#### Internalizing and externalizing mental illness symptoms

Mental health problems, both externalizing and internalizing symptoms, were assessed with the self-report form of the Strength and Difficulties Questionnaire (SDQ, [31]). We used the Hungarian version of the scale [32]. The instrument has 25-item allotted into five subscales: Emotional symptoms, Conduct problems, Hyperactivity/inattention, Peer relationship problems and Prosocial behaviour. A total difficulties score can be computed according to the first 4 factors. Participants were asked to score the items on a scale from 0 to 2 (0 = Not true, 1 = Somewhat true, 2 = Certainly true). Higher scores on the first four subscales (symptomatic scales) indicate more severe problems, while on the Prosocial behaviour subscale the higher ratings refer to more prosocial activities. The internal consistency of the total symptoms scale as measured by the Cronbach alpha test was adequate (α = .75). In addition, two of the subscales (emotional symptoms = .68 and prosocial behaviour = .64) were satisfactory. However scores for hyperactivity/inattention (.59), peer relationship problems (.54) and conduct problems (.45) were seen as only questionable or poor. Internal consistency score compare well with the original questionnaire (see [31]).

### Data analysis

The present study conducted variable- and person-oriented analyses related to the well-being dimensions measured by the MHC-SF. Firstly, a series of confirmatory factor analyses (CFA) and exploratory structural equation modeling (ESEM) were performed to evaluate the degree of model fit. Building on the findings of previous studies as described above six competing measurement models were compared: (1) a single-factor CFA model, (2) a two-factor CFA model, (3–4) a three-factor model using CFA and ESEM separately, and (5–6) a bifactor model specified separately in a CFA and ESEM framework.

The assessment of the measurement models was performed by using Weighted Least Squares Mean and Variance (WLSMV) adjusted estimation. The second step of the analysis tested the direct effects of gender and age on the latent factors of the best fitting model using a Multiple Indicator Multiple Causes (MIMIC) model. Furthermore, the assumptions of configural, metric and scalar invariance were also analysed for the best fitting model in a multiple group analysis between boys and girls.

Latent profile analysis (LPA) was conducted to identify latent classes of participants based on their well-being profile characteristics. The average item scores of the three subscales of well-being were used as continuous indicator variables. Models containing increasing numbers of latent classes were estimated. An analysis of multinomial logistic regression was performed to explore the relationship between the most likely latent class membership and covariates. The effect of age, gender, performance in school, family wealth, loneliness, and the subscales of the SDQ were analyzed.

Further details related to the CFA, ESEM and LPA model specification are presented in the Supplementary material.

IBM SPSS Statistics Version 25.0 and Mplus Version 8.1 [33] statistical softwares were used for the analysis.

## Results

### Factor structure of the MHC-SF

Table 1 displays the model fit indices related to the one-, two-, three-factor and bifactor model of the MHC-SF. Each of the measurement models showed significance according to the χ^2^-test, which indicated inadequate model fit. However, according to less conservative fit measures, among both CFA and ESEM models a bifactor structure of the well-being items yielded the closest fit to the data. In order to select the best fitting model, Model 5 and Model 6 were contrasted in terms of model fit. The deviation in the value of CFI and RMSEA was considered between the two models, using recommendations by Chen [34]. The value of ΔCFI and ΔRMSEA were below .01 and .015, respectively, therefore the fit indices didn’t indicate salient differences between these models. The more parsimonious and restrictive Model 5 (e.g., cross-loadings were fixed at 0) was retained and selected for further analyses [35].

**Table 1:** Degree of model fit and measurement invariance of the competing models

	χ^2^	df	RMSEA	Cfit of RMSEA	CFI	TLI	Δχ2	Δdf	ΔRMSEA	ΔCFI	ΔTLI
**General model fit of the measurement models**											
*Model 1*: One-factor model (CFA)	1360.98	77	.103	<.001	.881	.859					
*Model 2*: Two-factor model (CFA)	1010.80	76	.089	<.001	.913	.896					
*Model 3*: Three-factor model (CFA)	532.12	74	.063	<.001	.957	.948					
*Model 4*: Three-factor model (ESEM)	318.51	52	.057	.023	.975	.957					
*Model 5*: Bifactor model (CFA)	336.07	63	.053	.208	.975	.963					
*Model 6*: Bifactor model (ESEM)	224.07	41	.053	.196	.983	.962					
**Comparison of the measurement models**											
Model 3 versus Model 5							228.61	11	.010	.018	.015
Model 4 versus Model 6							119.25	11	.004	.008	.005
Model 3 versus Model 4							265.53	22	.006	.018	.009
Model 5 versus Model 6							161.87	22	.000	.008	- .001
**Model fit in each group separately (Model 5: Bifactor model, CFA)**											
Boys	197.70	63	.053	.272	.971	.958					
Girls	174.13	63	.047	.715	.983	.975					
**Measurement invariance testing (Model 5: Bifactor model, CFA)**											
Configural invariance	370.85	126	.050	.506	.978	.968					
Metric invariance	388.20	150	.045	.928	.978	.974					
Scalar invariance	521.77	202	.045	.958	.971	.974					
Configural versus metric invariance							58.29	24	.005	.000	.006
Metric versus scalar invariance							200.92	52	.000	.007	.000

*χ*^*2*^ Chi Square test statistics, *RMSEA* Root Mean Squared Error of Approximation, *Cfit of RMSEA* Closeness of fit test related to RMSEA, *CFI* Comparative Fit Index, *TLI* Tucker-Lewis Index, *SRMR* Standardized Root Mean Square Residual; *Δχ*^*2*^ Chi Square difference test. Chi Square test statistics and Chi Square difference test statistics are significant at least *p* < .05 level

Table 2 provides the standardized factor loadings and reliability indices related to Model 5. The values of the reliability indices indicated adequate internal consistency.

**Table 2:** Standardized factor loadings and reliability indices of the bifactor CFA model (Model 5)

Items	GWB^a^	EWB^b^	SWB^c^	PWB^d^
1	**.60**	**.44**		
2	**.67**	**.60**		
3	**.75**	**.28**		
4	**.48**		**.14**	
5	**.38**		**.14**	
6	**.56**		**.60**	
7	**.58**		**.49**	
8	**.51**		**.49**	
9	**.72**			**.20**
10	**.57**			**.13**
11	**.44**			**.17**
12	**.47**			**.30**
13	**.50**			**.61**
14	**.64**			**.42**
ECV^e^	67.3%	9.3%	12.9%	10.6%
Omega	.91	.85	.78	.81
Omega hierarchical	.80	.26	.27	.19
Relative Omega^f^	87.9%	30.6%	34.6%	23.5%
H	.88	.47	.55	.50
PUC	.69			

Factor loadings presented by bold figures are significant at least *p* < .001 level

^a^ General well-being. ^b^ Emotional well-being. ^c^ Social well-being. ^d^ Psychological well-being. ^e^ Explained Common Variance (ECV). ^f^ Relative Omega = Omega hierarchical / Omega. *H* H-index, *PUC* Percentage of uncontaminated correlations

A high proportion (67.3%) of the common variance was attributable to the general well-being factor, while specific well-being factors explained much lower rates (9.3–12.9%). The general and specific scale score variance were explained to a large extent by the combination of specific and general factors (78–91%). However, when taken alone specific factors only accounted for between 19 and 27% of the subscale variance attributable to the underlying target construct.

The measurement invariance of the MHC-SF were tested across both gender groups based on Model 5. Model fit results related to the different levels of equality constraints are presented in Table 1. The χ^2^ test showed significant result for each of the invariance models. However, the values of less conservative fit indices displayed acceptable fit for the three levels of measurement invariance models. The change in the values of CFI and RMSEA was considered between the invariance models, using recommendations by Chen [34]. As a consequence of the increasing level of equality constraints, the values of ΔCFI and ΔRMSEA were below .01 across the different levels of measurement invariance. Therefore, more liberal fit measures implied that the factor loadings and thresholds are invariant between boys and girls.

In the MIMIC model the effects of age and gender were examined on the latent factors specified by Model 5. The results of the bifactor CFA model with covariates are displayed in Table 3. Girls were more likely to report lower levels of emotional, social and psychological well being, but the direct effect of gender on the overall general well-being factor was not significant. Age showed significant negative relationship with the general well-being factor, and the specific factors of emotional and social well-being.

**Table 3:** Standardized regression coefficients of the covariates predicting well-being factors (CFA with covariates, Model 5)

		Dependent variables (Latent Factors)			
		General well-being	Emotional well-being	Social well-being	Psychological well-being
Covariates	Age	**−.17**	**−.19**	**−.36**	**−**.01
	Gender^1^	.02	**−.27**	**−.12**	**−.18**

All figures are standardized regression coefficients. Estimates presented with bold figures are significant at least *p* < .001 level. Model fit indices: χ^2^(83) = 438.88, *p* < .001; CFI = .966; TLI = .951; RMSEA = .052; ^1^Gender: 1 = Boys, 2 = Girls

### Latent profile Analysis (LPA)

Models containing two to five latent classes were estimated and compared. Table 4 presents the fit indices related to the models with an increasing number of latent classes. An improvement was demonstrated in the values of AIC, BIC, SSA-BIC and Entropy between models with three to five latent classes. However, in case of the five-class solution the result of the LMRT was non-significant compared to the model with four latent classes. This provided some indication that the inclusion of an additional latent class didn’t provide significant improvement in the model fit. Therefore, a model with four latent classes was retained and selected for further analysis.

**Table 4:** Fit indices for the latent class analysis of the well-being factors

	AIC	BIC	SSA-BIC	Entropy	LMRT	p
2 class model	12,246.67	12,300.19	12,268.43	.751	1016.84	<.001
3 class model	11,969.19	12,044.13	11,999.65	.682	276.09	<.001
4 class model	11,895.67	11,992.02	11,934.83	.686	78.84	= .001
5 class model	11,830.52	11,948.27	11,878.38	.690	70.75	= .193

*AIC* Akaike Information Criteria, *BIC* Bayesian Information Criteria, *SSA-BIC* Sample Size Adjusted Bayesian Information Criteria, *LRT* Lo-Mendel-Rubin Adjusted Likelihood Ratio Test

**Fig. 1:**
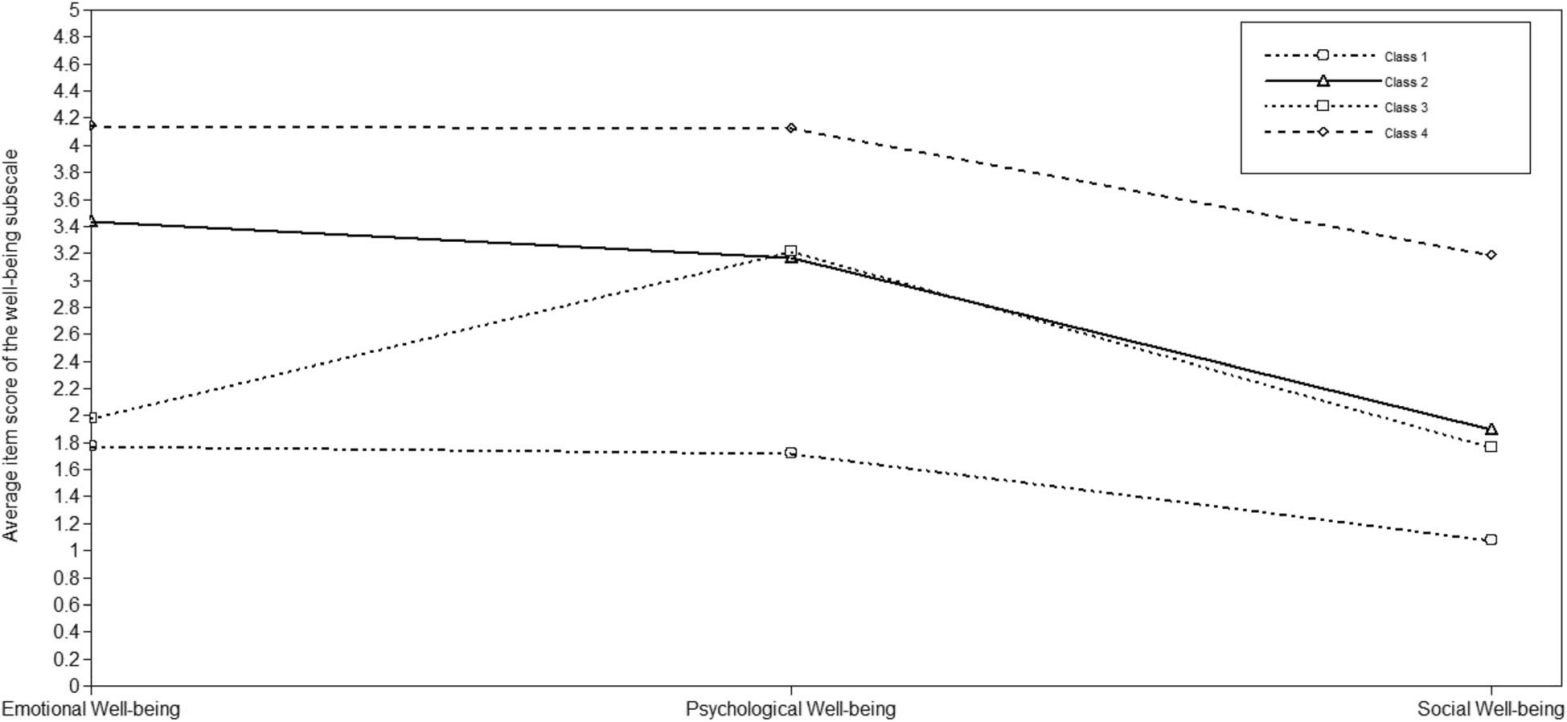
Latent class profiles related to the three well-being dimensions

Partially based on Keyes’ classification [1] the four latent classes were labelled as *Languishing* (Class 1), *Moderate Mental Health* (Class 2), *Emotionally Vulnerable* (Class 3), and *Flourishing* (Class 4) subgroups. The average latent class probabilities for the most likely latent class membership were 0.85, 0.79, 0.69 and 0.86, respectively. The profile characteristics of the four subgroups based on the average item scores of the three well-being subscale are illustrated in Fig. 1 and Supplementary Table 1.

Further analysis were conducted to explore the relationship between the identified latent classes and important covariates during adolescence, namely the effects of age, gender, performance in school, family wealth, loneliness, emotional symptoms, conduct problems, hyperactivity, peer problems and prosocial behavior. The results of the multinomial regression analysis are summarized in Table 5. Compared to the Languishing latent class (the reference category), higher levels of prosocial behaviour significantly predicted the membership to the Emotionally Vulnerable subgroup. In case of the Moderate Mental Health latent class, girls had lower probability getting into the class, performance in school and prosocial behaviour presented a significant positive relationship, while loneliness and peer problems had a significant negative relationship with the class membership relative to the Languishing subgroup. Finally, younger age, boys, lower levels of loneliness, emotional symptoms and peer problems, and elevated rates of school performance, family wealth and prosocial behaviour significantly predicted the membership of Flourishing class compared to the Languishing class.

**Table 5:** Odds ratios (95% Confidence Intervals) of the association between validating covariates and latent class membership relative to the Languishing subgroup (Class 1)

	Emotionally Vulnerable (Class 3) OR [95% CI]	Moderate Mental Health (Class 2) OR [95% CI]	Flourishing (Class 4) OR [95% CI]
Age	1.03 [.84–1.27]	.94 [.84–1.05]	**.75 [.65–.86]**
Gender^1^	.93 [.37–2.34]	**.37 [.22–.63]**	**.52 [.28–.95]**
Performance in school^2^	.91 [.44–1.88]	**1.69 [1.16–2.46]**	**2.55 [1.61–4.02]**
Family wealth^3^	.94 [.46–1.94]	1.20 [.85–1.69]	**1.68 [1.10–2.58]**
Loneliness^4^	.83 [.51–1.35]	**.39 [.28–.54]**	**.16 [.10–.28]**
Emotional symptoms^5^	.94 [.78–1.13]	.95 [.84–1.07]	**.74 [.62–.88]**
Conduct problems^5^	1.17 [.88–1.55]	1.12 [.94–1.33]	1.09 [.87–1.37]
Hyperactivity^5^	.91 [.74–1.13]	.95 [.84–1.09]	.91 [.77–1.06]
Peer problems^5^	.84 [.67–1.05]	**.81 [.71–.92]**	**.66 [.55–.80]**
Prosocial behavior^5^	**1.91 [1.47–2.47]**	**1.36 [1.17–1.57]**	**2.13 [1.73–2.62]**

Odds ratios presented in bold are significant at least *p* < .05 level. ^1^ Gender: 1 = Boys, 2 = Girls; ^2^ Performance in school: ordinal scale from 1 (among students with below average performance) to 4 (among students with the best performance); ^3^ Family wealth: ordinal scale from 1 (not wealthy at all) to 5 (very wealthy); ^4^ Frequency of loneliness: ordinal scale from 1 (no, never) to 4 (very often); ^5^ Subscales of the Strength and Difficulties Questionnaire (SDQ)

## Discussion

The importance of identifying and analysing the constructs of positive psychology in younger age-groups is twofold. Firstly, it can inform the enhancement of psychological practice and prevention. It can also however, improve social dialogue to encourage positive approaches to development. This study aims to support this endeavour by testing competing factor solutions of the Adolescent MHC-SF scale. In doing so the intent was to produce a set of well-being profiles and their characteristics in a large adolescent sample.

According to our results the factor structure of the Adolescent MHC-SF is best described by a bifactor model (similar to findings by Longo [19] and Rogoza [20]). This infers that the measured construct has a dominant global factor (general well-being) and specific components (emotional, psychological, and social well-being). It is also important that the variance of both the total well-being score and the underlying specific factors can be explained the best by the general and the specific health factors together. These results show that we can consider the general factor (i.e. the total score) as an indicator of overall well-being, which can comprise both the emotional, the psychological and the social well-being domains of the positive human life. At the same time, the specific components cover the multidimensionality of positive mental health, in line with the health definition of the World Health Organization [36]. Thus, our results support the multidimensional factor structure proposed by Keyes [6] and highlight that both the general well-being scale and the three subscales of the measurement fit in well with the need of the detailed evaluation and diagnostic process of adolescent mental health (cf. [37]).

Measurement invariance was supported across gender, suggesting that any gender differencies found for males and females when using the MHC-SF are not an artefact. All types of equivalence (configural, metric, scalar) were strenghtened, suggesting that boys and girls attribute the same meanings to questionnaire items, interpret the underlying latent construct in the same way. This is in line with results from previous studies’ [11, 15, 16, 20].

Our research detected significantly lower emotional, psychological, and social well-being among girls when analysed separately, but there was no gender difference in general well-being. This contrasts with findings by Rogoza’s [20], who revealed higher general well-being among males. That said, in this study the sample included adolescents, students, and adults. With respect to, we found that younger teenagers have better general well-being, in addition to higher emotional and social welfare. However, in our study, psychological well-being seemed to be independent of age, possibly due to the ongoing characteristics of puberty during adolescence. For example, adolescents have to continually deal with normative developmental tasks such as becoming more autonomous, working out individual identity, managing relationships and creating principle values in their life [38]. All of these challenges are inherent components of psychological well-being and can be assessed by the MHCM [1]. Declines in emotional, social, and general well-being during adolescence are consistent with the data which reveal growing emotional and social problems, mainly anxiety and mood disorders during late adolescence [39]. This is understandable given the ongoing neurological and hormonal changes leading to increases in emotional volatility and impulsivity (c.f [40].). These can reduce positive emotions and satisfaction with life together with social capacity.

The main novelty of our research was to generate positive mental health profiles in a large adolescent sample using non theoretical techniques. We performed LPA to identify subgroups with different well-being profiles and to compare these classes with Keyes’ original classification [1]. The LPA revealed four well-interpretable subgroups. Around 14% of adolescents had low values for all three well-being domains. This subgroup also had the highest rates of peer problems, loneliness, worst school performance and lowest prosocial behaviour. They could therefore be characterized as being at risk. This sub-group can be clearly identified as aligned to Keyes’ languishing group [6]. Thirty nine percent of the sample had average emotional and psychological well-being with lower social prosperity but average social well-being higher than in the languishing group. This group is equivalent to moderate mental health in Keyes’ classification. In a third detected subgroup, 9.8% of the adolescents, reported moderate psychological well-being with similar level of social well-being as in the second group, but low emotional well-being. The second and the third subgroups only vary in the level of emotional well-being, therefore we called the third subtype emotionally vulnerable. This newly identified group with decline in happiness and satisfaction with life and increment in negative feelings shows higher prosocial behaviour than the languishing group. Higher levels of prosociality in the emotionally vulnerable group may indicate a rationale for the higher levels of psychological well-being. Behaviours that are intended to benefit others could enhance the own psychological and social well-being such as environmental mastery or having positive relation with others [2]. Our findings can even better specify and elaborate on Keyes’ original conception about the languishing and moderate categories. Finally, a strong fourth subset of positive mental health emerged during the analysis showing high levels of emotional and psychological well-being together with an already good social well-being in this age. Thirty six percentage of the sample fell into this subtype. This cluster is particularly compatible to Keyes’ flourishing group [6]. He found a similar distribution of flourishing adolescents in an American sample (37.9%) [6]. The flourishing adolescents in our sample also had low internalizing problems (c.f [6, 17].).

On the whole, the four latent profiles showed different patterns of association with mental illness symptoms and sociodemographic data. Higher SES, better school performance, more prosocial acts, lower loneliness, and emotional and peer problems, male gender, younger age predicted the membership of the flourishing class contrasted to the languishings. Behavioural problems did not distinguish membership of the classes, but this result may due to the lower internal consistency of these subscales.

The detected latent mental health profiles in our study further support the application of the self-report version of Adolescent MHC-SF, which has proven to be an effective positive mental health detector throughout the preiod of adolescence. However, the original classes of the MHCM, which are based on theoretical basis, may need refinement.

### Limitations and further objectives

This study examined a generally healthy population. Additional research should test the examined models in populations at either end of the functioning spectrum. For example, with adolescent psychiatric patients and elite athletes to determine whether the latent profiles are replicable. It may be that these populations exhibit different profiles [41]. It would also be useful to include other variables, such as earlier stressful or important positive life events; peer, parental and family influences to see their impact on positive mental health profiles. The analyses carried out in this study could also be replicated in emerging adult and adult populations to build up a set of well-being profiles across the life course.

## Conclusions

By uncovering latent subtypes of positive mental health this study can contribute to the refinement of the Mental Health Continuum Model. Besides languishing, moderate mental health, and flourishing profiles, an emotionally vulnerable subgroup was found, suggesting the levels of mental health are more finely nuanced.

## Supplementary information

**Additional file 1.** Supplementary material [34, 35, 42]

## Data Availability

The dataset used and analysed during the current study is available from the corresponding author on reasonable request.

## Abbreviations

AIC: Akaike Information Criteria
BIC: Bayesian Information Criteria
Cfit of RMSEA: Closeness of fit test related to RMSEA
CFA: Confirmatory factor analyses
CFI: Comparative Fit Index
ECV: Explained Common Variance
ESEM: Exploratory structural equation modeling
H: H-index
LPA: Latent Profile Analyses
LRT: Lo-Mendel-Rubin Adjusted Likelihood Ratio Test
MDS: Multidimensional scaling
MHCM: Mental Health Continuum Model
MHC-SF: Mental Health Continuum Short Form
MIMIC: Multiple Indicator Multiple Causes
PUC: Percentage of uncontaminated correlations
RMSEA: Root Mean Squared Error of Approximation
SSA-BIC: Sample Size Adjusted Bayesian Information Criteria
SDQ: Strength and Difficulties Questionnaire
SRMR: Standardized Root Mean Square Residual
TLI: Tucker-Lewis Index
WLSMV: Weighted Least Squares Mean and Variance
χ^2^: Chi Square test statistics
Δχ^2^: Chi Square difference test
