# Supplementary material for: Well-being profiles in adolescence: psychometric properties and latent profile analysis of the mental health continuum model – a methodological study
Source: Health Qual Life Outcomes. 2020 Apr 6;18:95. doi: 10.1186/s12955-020-01332-0 (PMC7137408; doi:10.1186/s12955-020-01332-0)
Supplement: Supplementary file 1 — Additional file 1. Supplementary material [34, 35, 42] [file 12955_2020_1332_MOESM1_ESM.docx]

**Supplementary material**

*Factor structure of the MHC-SF*

The present study assessed the model fit of six competing measurement models. Model 1 presented a single-factor concept of well-being, where all the items of the MHC-SF were loaded on the general factor of well-being. Model 2 contained the correlated factors of hedonic and eudaimonic well-being. The items of the eudaimonic well-being factor (4-14) covered the construct of psychological and social well-being. Model 3 assessed the correlated dimensions of emotional, social and psychological well-being. Model 5 referred to a bifactor model of well-being, which measures three specific and one general, orthogonal factors of well-being on the same conceptual level. Each of the items of the MHC-SF were simultaneously loaded on one of the specific well-being (emotional, psychological and social) factors and on the overall factor of general well-being. These models were specified in a CFA framework and the cross loadings were fixed at 0. Model 4 and Model 6 estimated a three-factor and a bifactor concept of well-being by using the ESEM approach. In the case of ESEM models, the application of target rotation allows the examination and to confirm a priori factor structure [35]. To obtain a more realistic measurement structure, factor cross loadings were estimated and set out to be close to 0. The present analyses defined the items of the MHC-SF as ordered categorical variables. The Model estimations also took into account the clustered sampling (within class) nature of the data.

Various model fit indices were considered in the process of model evaluation. For the Comparative Fit Index (CFI) and Tucker-Lewis Index (TLI) a score of around .90-.95 indicates an acceptable fit. In the case of adequate fit, the value of Root Mean Squared Error of Approximation (RMSEA) should be below .05, and the result of the Closeness of fit test (Cfit of RMSEA) should show a non-significant result (p>.05) [42].

For the best fitting measurement model the assumptions of configural, metric and scalar invariance were also tested in a multiple group analysis between boys and girls. The hypothesis of configural invariance, the factor loadings and thresholds were freely estimated during the model testing. At the level of metric invariance the factor loadings were estimated at equal across the two groups. Based on the assumption of the scalar invariance of the measurement model, the equality of the factor loadings and thresholds were considered across the two groups. To evaluate the level of invariance of the measurement model, the change in the values of CFI and RMSEA was considered between the invariance models based on the recommendation by Chen [34]. A deviation in the value of CFI and RMSEA below .01 and .015, respectively, between the measurement invariance models indicates invariance across the groups [34].

*Latent profile analysis (LPA)*

Latent profile analysis (LPA) was conducted to identify subgroups of participants based on their well-being profile characteristics. During an iterative model estimation, LPA models with growing number of latent classes were tested, starting with the most parsimonious two-class solution. Models were compared along multiple fit indices. In the case of the Akaike Information Criterion (AIC), Bayesian Information Criterion (BIC), and Sample Size Adjusted Bayesian Information Criterion (SSA-BIC) – as the measures of parsimony of each model – lower values imply more sufficient model fit relative to models with a different number of latent classes. The index of Entropy with higher values (e.g. closer to 1) indicates more accurate classification of the participants. Significant result (p<.05) of the Lo-Mendel-Rubin Adjusted Likelihood Ratio Test (LMRT) presents more adequate model fit of the estimated model by involving an additional latent class compared to the previous model with lower number of latent classes. In the case of a non-significant LMRT result, further LPA models were not assessed, as the data indicated that the involvement of an additional latent class didn’t provide significant development in the model fit.

Supplementary Table 1 Average item scores on the three well-being subscales of the identified latent classes

|  | Flourishing class (N=572; 36.7%)  M (S.E.) | Moderate Mental Health class (N=610; 39.1%)  M (S.E.) | Emotionally Vulnerable class (N=153; 9.8%)  M (S.E.) | Languishing class (N=225; 14.4%)  M (S.E.) |
| --- | --- | --- | --- | --- |
| Emotional well-being | 4.15 (.04) | 3.44 (.07) | 1.98 (.13) | 1.77 (.10) |
| Psychological well-being | 4.13 (.04) | 3.17 (.10) | 3.21 (.17) | 1.72 (.10) |
| Social well-being | 3.18 (.10) | 1.89 (.07) | 1.76 (.11) | 1.07 (.06) |
